# Fetal Membranes Contribute to Drug Transport across the Feto-Maternal Interface Utilizing the Breast Cancer Resistance Protein (BCRP)

**DOI:** 10.3390/life12020166

**Published:** 2022-01-23

**Authors:** Ananthkumar Kammala, Meagan Benson, Esha Ganguly, Enkhtuya Radnaa, Talar Kechichian, Lauren Richardson, Ramkumar Menon

**Affiliations:** Department of Obstetrics & Gynecology, Division of Basic and Translational Research, The University of Texas Medical Branch at Galveston, Galveston, TX 77555, USA; ankammal@utmb.edu (A.K.); mebenson@utmb.edu (M.B.); esgangul@utmb.edu (E.G.); enradnaa@utmb.edu (E.R.); takechick@utmb.edu (T.K.); lestaffo@utmb.edu (L.R.)

**Keywords:** transport protein, drug transport, pregnancy, exosomes

## Abstract

During pregnancy, the placenta is established as a primary organ for drug transport at the maternal-fetal interface. The fetal membranes (FM) also form an interface with maternal tissues; however, their role in drug transport has not been previously investigated. Knowledge of drug transport across this feto-maternal interface along with the placenta can improve new drug development and testing for use during pregnancy. We also hypothesize that extracellular vesicles (exosomes 30–160 nm) released from the FM and placental cells may also contain drug transport proteins and might impact drug trafficking across the feto-maternal interfaces. The objectives were to (1) localize the breast cancer resistance protein (BCRP) in human FM; (2) determine the drug transport function of BCRP in chorion trophoblast cells (CTCs) of the FM; and (3) investigate the presence of BCRP in FM cell-derived exosomes, as a paracrine modifier of the tissue environment for transport functions. The gene and protein expressions of ABCG2/BCRP in FMs were determined by quantitative real-time PCR (qRT-PCR) and western blotting (WB) and were localized by immunohistochemistry (IHC). The surface expression of BCRP in FM cells was determined by flow cytometry. The functional role of BCRP was assessed by an EFFLUX dye multidrug resistance assay. The presence of BCRP in exosomes derived from CTCs and BeWo cells was examined using ExoView^®^. Data derived from CTCs are compared with placental trophoblast cells (BeWo). BCRP is expressed and localized in the fetal membrane, primarily in the chorion trophoblast cell layer and scarcely in the amnion epithelial layer (AEC), and primarily localized on both AEC and CTC cell surfaces. Efflux assay data showed that FM cells have similar drug resistance activity as BeWo cells, suggesting that FM also have drug transportation capabilities. BeWo- and CTC-derived exosomes expressed limited BCRP protein on the surface, so it was predominantly contained in the exosomal lumen. As far as we are aware, this is the first study to report BCRP expression in fetal membrane cells and as cargo in fetal membrane-derived exosomes. We report that fetal membrane cells are capable of drug transportation. Based on these results, investigational drug trials should include the FM and its exosomes as possible drug transportation routes in pregnancy.

## 1. Introduction

Preterm birth (PTB) is a complex obstetric complication that leads to significant morbidity and mortality despite decades of research [1]. Various progesterone preparations have been utilized to reduce the risk of spontaneous PTB; however, data regarding the route of administration, dosage, and efficacy are not consistent. [2,3,4] These inconsistencies in data have led to variations in the current clinical practice regarding the prevention of spontaneous PTB [5,6,7]. A better understanding of the pharmacokinetics in pregnancy is needed to improve prevention strategies and treatment for spontaneous PTB and other medical conditions during pregnancy. 

The study of pharmacotherapy in pregnancy has significant pragmatic and ethical limitations, making it difficult to establish a standard of care regarding the prediction, prevention, and treatment of complications during pregnancy, such as PTB. Pharmacokinetics involves the examination of the absorption, distribution, metabolism, and excretion of a drug. Unique physiological changes during pregnancy alter drug pharmacokinetics [8]. Drug transport proteins are responsible for the movement of drugs between compartments and across barriers. The feto-maternal interface serves as a barrier between the pregnant patient and the fetus, which helps to maintain pregnancy, and includes the transfer of gases, nutrients, and immune cells as well as the removal of harmful xenobiotics. Previous studies have examined the role of multiple drug transport proteins like P-glycoprotein (P-gp), breast cancer resistance protein (BCRP), multidrug resistance-associated proteins (MRP1, MRP2, MRP3, MRP4, and MRP5), organic anion-transporting polypeptides (OATP4A1, OATP1A2, OATP1B3, and OATP3A1), organic anion transporter 4 (OAT4), organic cation transporter 3 (OCT3), and organic cation/carnitine transporters (OCTN1 and OCTN2), in the placenta [9,10]. Our recent data showed that fetal membranes (FMs—amniochorion), along with the placenta, express various transport proteins; however, their localization within FM cells and functions have not yet been elucidated. Since human fetal membranes are involved in various protective mechanisms [11,12,13,14,15,16] during pregnancy, it is likely that transport protein functions may also be controlled by the FMs. Determining the role of FMs in drug transport is necessary and could lead to a greater understanding of pharmacokinetics in pregnancy. 

Human FMs are comprised of the amnion epithelial cell (AEC) and chorion trophoblast cell (CTC) layers. AECs are in contact with the amniotic fluid, while the CTCs line up with maternal decidua (DEC), forming an important connection between a pregnant patient and the fetus. The FM-decidual interface forms the second barrier, similar to the placental-decidual barrier [15]. We hypothesize that the fetal membranes serve as a gatekeeper for drug transportation by utilizing drug transporter proteins. Establishing an alternative route for drug transportation between the mother and developing fetus could lead to substantial advancement in the study of pharmacokinetics in pregnancy and ultimately contribute to the generation of vital information needed for preclinical and clinical trials. 

The present study focuses on the human breast cancer resistance protein (BCRP/ABCG2), an ATP-binding cassette efflux transporter protein previously identified in the placenta [10,17]. BCRP is a well-expressed drug transport protein throughout the human body, including the apical surface of the placental syncytiotrophoblasts, liver hepatocytes, the intestinal tract epithelium, the endothelial cells of the brain, and the proximal renal tubules [18]. BCRP contributes to the distribution and elimination of drugs and other substances, both endogenous and exogenous; therefore, BCRP is thought to confer cellular protection against xenobiotic exposure. The United States Food and Drug Administration (FDA) recognizes BCRP as clinically relevant transporters involved in drug disposition [18]. In addition, the expression of BCRP in the placenta has been shown to decrease with advancing gestation [9]. These points further support the need to investigate the expression of BCRP in the FMs as this mechanism is potentially a critical missing component in our current understanding of drug transportation at the feto-maternal interface. 

Another important area of study is determining the potential paracrine role that extracellular vesicles (exosomes of 40–160 nm) contribute to drug transportation and the protection of cells from xenobiotics. Exosomes are membrane-bound extracellular vesicles that play a significant role in communication and signaling at the feto-maternal interface [19]. Exosomes carry important cargo between their cells of origin and target cells. Multiple studies performed in our laboratory have demonstrated the potential functional role of exosomes derived from FM cells [20,21,22]. Exosomes derived from the AEC layer have been shown to carry important cargo that reflect the status of the cell in an oxidative stress environment [21]. In addition, AEC-derived exosomes have led to an increased inflammatory response in maternal uterine cells, which could potentially play an important role in parturition [20,22]. We hypothesize that FM cells express BCRP and exosomes derived from FM cells contain the BCRP transport protein and potentially play a role in drug transportation across the feto-maternal interface. Exosomes derived from placental cells (BeWo) were used as a control. 

## 2. Materials and Methods

### 2.1. Tissue Collection and Institutional Review Board Approval

The collection of placentas was approved by the Institutional Review Board (IRB) at the University of Texas Medical Branch (UTMB) at Galveston, TX (IRB approval number IRB16.0058, January 2020). The protocol allows for the collection of discarded placentas from term vaginal or term, not in labor, cesarean deliveries (TL and TNIL, respectively) from John Sealy Hospital according to our laboratory’s inclusion and exclusion criteria as described in previous reports [13,14]. The protocol is exempt from subject consent as the specimens are non-identifiable and were intended to be discarded. 

### 2.2. Tissue Processing 

Placentas and fetal membranes were processed according to our laboratory’s protocol for collection, which has previously been described [14,23,24]. Briefly, the FM were dissected from the placenta and 6-mm biopsies were performed from the midzone of the FM for explant preparation for WB. FMs and placentas were fixed for 48 h in 4% paraformaldehyde and embedded in paraffin for immunohistochemistry (IHC). 

### 2.3. Quantitative Real-Time PCR for ABCG2 

A high-speed homogenizer dissociated FMs and placentas in Trizol (Life Technologies, Carlsbad, CA, USA). RNA was isolated using the RNeasy Kit (QIAGEN, Hilden, Germany) and treated with RNase-free DNase (QIAGEN). According to the manufacturer’s instructions, cDNA was synthesized using a High-Capacity cDNA Reverse Transcription Kit (Thermo Fisher, Waltman, MA, USA). According to the manufacturer’s instructions, real-time qRT-PCR was performed using a validated TaqMan gene expression ABCG2 primer (assay ID: Hs00188594_m1, gene symbol: ABCG2) probe sets. qRT-PCR reactions were carried out using Bio-Rad CFX PCR instruments (Bio-Rad, Hercules, CA, USA). Results were analyzed using CFX Maestro software. The gene expression was calculated using the 2−∆Ct method with GAPDH as an internal reference control.

### 2.4. Immunohistochemistry for BCRP Localization on the FM

IHC for BCRP was performed on the paraffin-embedded FM and placenta blocks prepared as above. Placental tissue served as a positive control as BCRP is known to be expressed in the placenta [10]. FMs without the application of the BCRP primary antibody served as the negative control. 

Five-µm tissue sections were cut and adhered to slides with a positive charge. Deparaffinization was performed with Xylene. Sections were rehydrated with 100% ethanol, 95% ethanol, and 70% ethanol. An IHC kit from Abcam (ab64264) was used and the manufacturer’s instructions were followed. An antigen retrieval system was used for epitope unmasking utilizing the Tris-EDTA buffer (pH = 9.0). Placenta and FM sections were incubated with a BCRP primary antibody (Abcam [ab3380]; 1:1000 dilution) at 4 °C overnight for immunostaining. For the negative control, FM sections were incubated overnight in 3% BSA TBS-T without a primary antibody. An IHC antirabbit antibody (1:500, Vector Laboratories, CA, USA) was added for 10 minutes at room temperature, followed by DAB as a chromogen and hematoxylin as a counter stain for color development. Slides were examined with bright field microscopy for the presence and localization of BCRP. Images were taken at 20× and 40× magnification. 

### 2.5. Culture of Immortalized Human FM Cells 

Maternal decidual and FM cells (AECs, amnion mesenchymal cells (AMCs), and CTCs) were isolated from term, not-in-labor, cesarean deliveries and immortalized by a standard HPV16 E6E7 retrovirus protocol [25]. AMCs and decidual cells (DECs) were cultured and maintained in complete media consisting of Dulbecco’s modified Eagle’s medium (DMEM) with a Ham F12 nutrient mixture 1:1, the antibiotics penicillin (100 IU/mL), streptomycin (100 µg/mL), and amphotericin B (2.5 µg/mL) and a 5% heat-inactivated fetal bovine serum. AECs were cultured in complete keratinocyte serum-free media (KSFM), a culture that is highly selective for epithelial cells, supplemented with a human recombinant epidermal growth factor (0.1 ng/mL), bovine pituitary extract (30 µg/mL), and primocin (0.5 mg/mL) (ant-pm-1; Invivogen). Supplements were added to the media immediately before use. CTCs were cultured in Dulbecco’s modified Eagle’s medium (DMEM) with a Ham F12 nutrient mixture 1:1 supplemented with the following: Fetal bovine serum, penicillin, streptomycin, bovine serum albumin, ITS-X, CHIR99021, A83-01, SB431542, L-ascorbic acid, epidermal growth factor, VPA, and Y27632 (ROCK inhibitor). Cells used for all experiments were within 10 passages. 

### 2.6. BeWo Cell Culture

BeWo cells (from ATCC, Manassas, VA, USA) were used to represent placental cytotrophoblasts. BeWo cells were plated in T75 flasks and maintained in DMEM and Ham’s F12 media with the addition of 10% HI-FBS, Amphotericin, and Penicillin/Streptomycin. The flasks were incubated at 37 °C, 5% CO2, and 95% humidity. The cells were grown to approximately 80% confluence and passed every 48–72 h. Cell culture media was changed every 48 h. 

### 2.7. Flow Cytometric Analysis of BCRP Expression

FM cell lines (5 x 10^5^ AECs, AMCs, CTCs, and DECs) were washed with cold FACS buffer (PBS containing 2% FBS) and stained with Phycoerythrin (PE) conjugated BCRP/ABCG2 Ab (BD Biosciences, San Jose, CA, USA) at 4 °C for 30 min in the dark. Cells were washed twice with cold FACS buffer and fixed in 250 mL of 1.5% formaldehyde in PBS. Samples were acquired on a LSR II flow cytometer (BD Biosciences) and analyzed using FlowJo software (FlowJo, Ashland, OR, USA). FM cells stained with PE-human IgG were used as an isotype control. The mean fluorescence intensity (MFI) of PE-BCRP was determined by FlowJo software (USA).

### 2.8. Protein Extraction and Western Blotting 

Placental and FM explant tissues and immortalized maternal decidual cells and FM cells (AEC, AMC, and CTC) were lysed with a radioimmunoprecipitation lysis buffer [50 mM Tris (pH 8.0), 150 mM NaCl, 1% Triton X-100, and 1.0 mM EDTA (pH 8.0), and 0.1% SDS] supplemented with a protease and phosphatase inhibitor cocktail and phenyl methyl sulfonyl fluoride. Human FMs were homogenized as previously described [13,14]. After centrifugation at 12,000 g for 20 min, the supernatant was collected and protein concentrations were determined using a Pierce bicinchoninic acid kit (Thermo Scientific, Waltham, MA, USA)]. The protein samples were separated using SDS-polyacrylamide gel electrophoresis on a gradient (4–15%) Mini-PROTEAN TGX Precast Gel (Bio-Rad, Hercules, CA, USA) and transferred to the membrane using a Bio-Rad Gel Transfer Device (Bio-Rad). Membranes were blocked in 5% non-fat milk in 1× Tris-buffered saline-Tween 20 for a minimum of 1 h at room temperature and then probed with the primary antibody overnight at 4 °C. The membranes were incubated with secondary antibody conjugated with horseradish peroxidase and immunoreactive proteins were visualized using the chemiluminescence reagent ECL WB detection system (Amersham Piscataway, NJ, USA). The following anti-human antibodies were used for western blot: BCRP (1:1000 for tissues and FM cells, ab245246, Abcam, CT, USA) and β-actin (1:15,000, Sigma-Aldrich, St. Louis, MO, USA). Protein bands were quantified against β-actin expression in each lane, and expressions were densitometrically determined using Bio-Rad Image Lab 6.0 software. 

### 2.9. eFluxx Dye Assay

The eFluxx dye assay (eFluxx-ID^®^ Green Multi-Drug Resistance (MDR) Assay Kit, ENZO Life Sciences, Inc., Farmingdale, NY, USA) was performed according to the manufacturer’s instructions [26]. Briefly, on the day of the assay, cells were collected, washed with PBS, and incubated with or without Novobiocin (BCRP inhibitor) in the presence of the eFluxx-dye for 30 min at 37 °C. eFluxx-dyes are xanthene-based small molecule dyes developed for the detection of MDR activity in living cells. To exclude dead cells in this assay, a propidium iodide (PI) solution was added to the cells during the last 5 min of incubation in some of the experiments. After incubation, cells were simply spun down (1 min, 200 g) to remove the excess probe, resuspended in an ice-cold medium, and kept on ice until flow cytometry was performed. The fluorescence intensity of eFluxx-IDH Green dye (ex/em 490/514 nm) was measured in the FL1/FITC (530/30 filter) channel. The unstained cells were shown as red, cells only treated with efflux dye were shown in green, and cells treated with a BCRP inhibitor and eFluxx dye were shown as blue. The mean fluorescence intensity (MFI) values were analyzed using FlowJo software. The MDR activity factor (MAF) of BCRP in different cell types was calculated according to the following formula:

MAF = 100 × (MFI with inhibitor − MFI control)/(MFI with inhibitor).

### 2.10. BeWo- and CTC-Derived Exosome Isolation and Purification

This study defines exosomes as extracellular vesicles with a particle size from 30–160 nm in diameter. Exosomes were isolated and purified by a process previously described by our laboratory [27,28]. Briefly, culture media was collected from BeWo and CTC cells following 48 h of cell use. The media was filtered and stored at −80 °C for use in future experiments. The frozen media was thawed overnight at 4 °C prior to the day of exosome isolation. The media were sequentially centrifuged at 4 °C at 300× *g* for 10 min and 2000× *g* for 20 min. Supernatants were concentrated in the Amicon^®^ ultra-15 centrifugal tube with a 100,000 nominal molecular weight limit (NMWL) for 90 min at 2000× *g* at 4 °C. Concentrated media was collected and then centrifuged at 10,000× *g* for 30 min at 4 °C. Supernatants were filtered through a 0.2-μm Nalgene™ syringe filter (Thermo Scientific, Waltham, MA, USA) and ultracentrifuged at 100,000× *g* in a type 70.1 Ti rotor (Beckman Coulter, Brea, CA, USA) for 2 h at 4 °C. The supernatants were then discarded and the pellets were resuspended in cold PBS. The Exospin™ exosome purification column was used to purify the exosomes. The exosomes were aliquoted and stored at −80 °C for further use. 

### 2.11. Determination of Exosome Size and Quantification

The size distribution and concentration of exosomes were determined using ZetaView™ PMX 110 (Particle Metrix, Meerbusch, Germany) and its corresponding software (ZetaView 8.02.28) as previously described by our lab [22,29,30]. Briefly, the frozen exosomes were thawed on ice and diluted in 1:1000 filtered distilled water. The number of particles/mL and size distribution were counted for each sample. The machine was cleaned between samples using filtered distilled water. The results of the ZetaView™ were used to calculate the number of exosomes per BeWo and CTC cell types.

### 2.12. ExoView Detection of BCRP

Exosomes were analyzed using the ExoView™ platform (NanoView, Boston, MA, USA) following the procedure established by the manufacturer with modifications. ExoView™ allows the detection of a specific cargo protein at a single-vesicle level. The protocol used in our laboratory has been previously reported [27]. Briefly, 35 μL of exosomes (1 × 10^9^ /mL) from BeWo and CTC cells were diluted according to the instructions and incubated on tetraspanin microarray chips placed in a 24-well plate at RT overnight. Each chip was pre-coated with CD9, CD63, and CD81 antibodies and MIgG control antibodies. The unbound exosomes were washed three times for 3 min in a 500-rpm shaker in solution A. Exosomes bound to the capture spots were then fixed and permeabilized with the ExoView™ cargo kit according to the manufacturer’s protocol. The bound exosomes were fixed with solution C (NanoView™ Biosciences) for 10 min, washed as previously described and lysed in solution D (NanoView™ Biosciences) for 10 min before being washed again as described. A PE-conjugated BCRP antibody (BioLegend ABCG2, Cat:332008) was used to determine the BCRP protein level in the isolated exosomes at a concentration of 0.3 μg per chip. Tetraspanin CD9 and CD63 antibodies were diluted in the same blocking solution (NanoView Biosciences) and were incubated with the exosomes for 1 h at RT in the dark. The tetraspanin microarray chips were then sequentially washed three times for 5 min in solution A and solution B (NanoView Biosciences) and five times for 5 min in Milli-Q water (ELGA) on a 500-rpm shaker. Chips were then imaged on the ExoView™ R100 instrument (NanoView™ Biosciences) using the nScan 2.9.3 acquisition software. The size distribution, concentration, and BCRP loading efficiency were calculated using NanoViewer 2.9.3 provided by NanoView™ Bioscience, and output was displayed and stored on an Excel spreadsheet. 

## 3. Results

### 3.1. Human FM Express BCRP Transport Protein 

**Figure 1:**
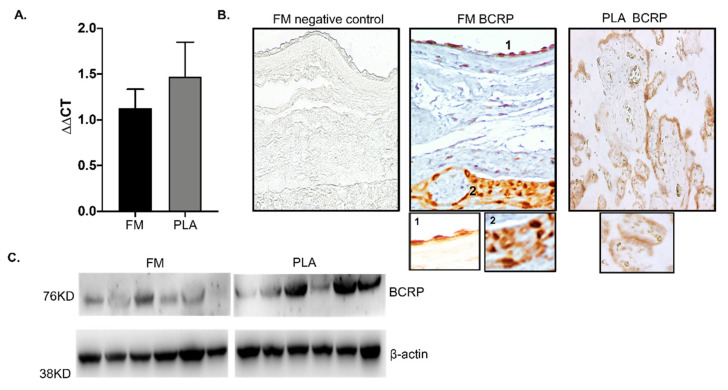
Expression and localization of ABCG2/Breast cancer resistance protein (BCRP) in human fetal membranes. (**A**) Real-time polymerase chain reaction (PCR) analysis of ABCG2 mRNA in the fetal membrane (FM) shows gene expression of ABCG2. The expression levels of ABCG2 are similar in both FM and placenta (PLA) tissues. (**B**) Representative immunohistochemistry images of FM and human stained for BCRP. BCRP was localized to both the amnion epithelial layer (1) and the chorion trophoblast layer (2) of the FM. The PLA staining serves as the positive control. (**C**) Western blots of BCRP in human FM compared to the PLA. Representative blots from *n* = 6 for each tissue type are shown. Data presented as mean ± SD.

Human FMs have morphologically distinct layers, each with a well-defined microarchitecture, including the amnion and chorion membrane [15,31]. To study gene expression and localize ABCG2/BCRP in the FM, we performed quantitative RT-PCR and IHC. The gene expression levels of ABCG2 in the FM were similar to those of the placenta (Figure 1A). BCRP was localized in the basal side of amnion epithelium (1) and predominantly expressed in CTCs (2) (Figure 1B). As reported in previous studies, and used here as a positive control (Figure 1B), BCRP was localized in the syncytiotrophoblast of the placenta, where it functions primarily to protect the developing fetus by effluxing the xenobiotics present back to the maternal circulation [10,17]. To confirm the relative expression levels of BCRP in FM, WB was performed. Confirming our previous data, BCRP was expressed in the FM as well as the placenta (Figure 1C). Since both the FM and placenta express BCRP, it is likely that both feto-maternal interfaces may perform drug transport functions during pregnancy. 

### 3.2. Human FM Trophoblasts Express More BCRP Than Placenta Trophoblasts

**Figure 2:**
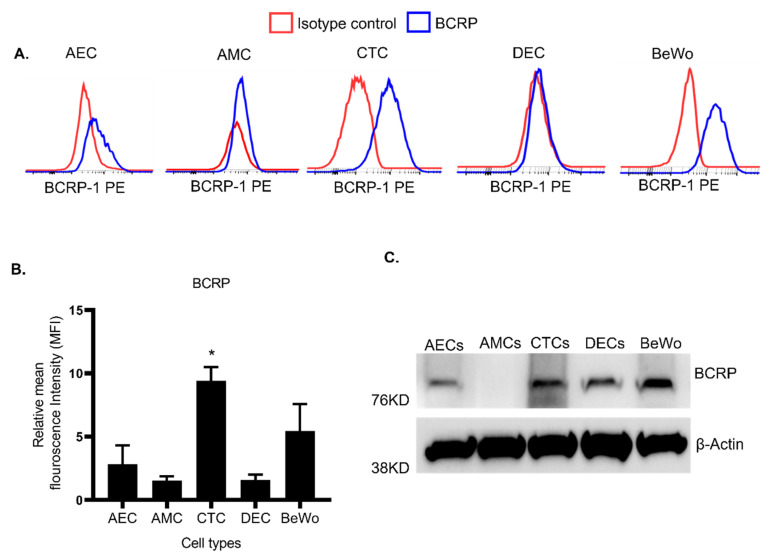
Expression of BCRP transport protein in human fetal membrane cell lines. (**A**) Flow cytometer analysis for the surface expression levels of BCRP in FM cell lines are shown: Amnion epithelial cells (AEC); amnion mesenchymal cells (AMC); chorion trophoblast cells (CTC); and decidua cells (DEC). Placental BeWo trophoblast cells were used as a positive control throughout these experiments. Cells stained with PE-Isotype control are indicated with red color lines and cells stained with PE-BCRP1 are indicated with blue color lines and demonstrate a distinct peak. (**B**) Relative mean fluorescence intensity (MFI) from different FM cells were calculated by FlowJo and are represented as a bar graph. CTCs showed a significant increase in BCRP expression compared to other cell lines. (**C**) Western blots of human FM cell lines for expression of BCRP. Representative blots of BCRP expression from each cell type are shown (*n* = 6). Data presented as mean ± SEM. Statistical significance was determined by an unpaired student *t*-test considering BeWo as a control. * *p* < 0.05.

We further evaluated the expression of BCRP in the FM cell lines. AEC, AMC, CTC, and decidua cell lines (DEC) were used to study the layers of the FMs and the maternal decidua. Placenta-derived BeWo cells were used as a control. FM and placenta cells were stained with PE-BCRP and subjected to flow cytometry to determine the surface expression levels of BCRP. The expression of PE-BCRP-stained cells has shown a greater shift of peaks from PE-Isotype-stained cells. The Isotype-stained cells are depicted as red peaks, whereas PE-BCRP-stained cells showing a distinct shift from isotype control were depicted as blue peaks in the flow charts. Consistent with IHC data, AECs and CTCs showed relatively higher expression levels of BCRP compared to isotype controls (Figure 2A). Interestingly, PE-BCRP-stained CTCs have shown a significant shift from the isotype control, compared to placenta BeWo cells. It indicates that BCRP expressed on the cell surface of CTCs was significantly (*p* < 0.05) higher than placenta BeWo cells (Figure 2A,B). WB data showed total BCRP expression in all FM cells, as well as AMCs (Figure 2C), suggesting that AMCs only have low levels of surface BCRP expression. These results confirm the differential expression of BCRP in FM cells. 

### 3.3. FM Cells Also Exhibit a Similar Efflux Activity as BeWo 

**Figure 3:**
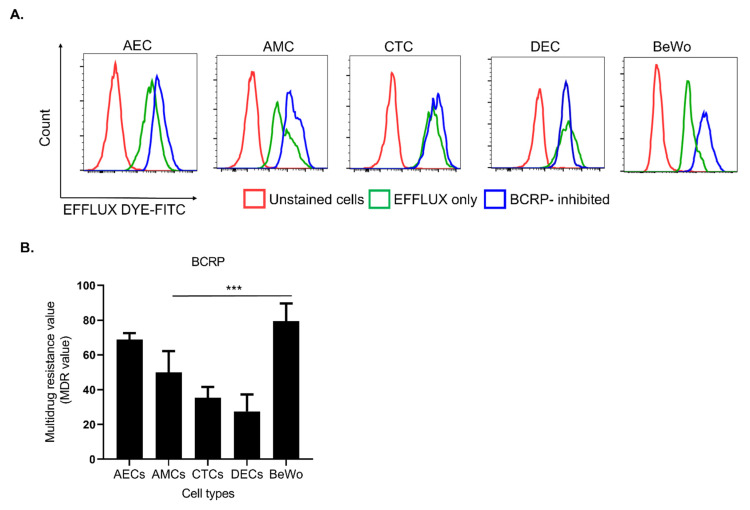
Functional assay of BCRP on FM cell lines using efflux dye kit. (**A**) To assess BCRP function in FM cell lines, an eFFlux dye study was conducted with and without an BCRP inhibitor (Novobiocin). Cells treated with only eFFlux dye (green peak) show a distinct peak from unstained cells (red peak). eFFlux dye diffused into the cells gets trapped by the inhibition of BCRP causing a greater shift (blue color). Cells with a higher amount of eFFlux dye will show greater fluorescence intensity, indicative of functionally active BCRP and cells with inactive BCRP allow efflux dye to drain out and express lower fluorescence, indicating lack of functionally relevant BCRP. Representative flow cytometer graphs of FM cell lines (*n* = 3) after eFFlux dye incubation. (**B**) The graph represents mean fluorescence intensity of the FM cells to determine a multidrug resistance activating factor (MDR). AECs that are directly in contact with amniotic fluid show a similar functional response as BeWo to efflux. Data represented as Mean ± SEM. Statistical significance was determined by an unpaired student *t*-test considering BeWo as a control *** *p* < 0.001.

Our data have shown that BCRP is expressed in FM cells along with placenta cells. To confirm that BCRP expression in FM cells contributes to the efflux drug transport mechanism, we performed the commercially available eFFlux dye assay. Specifically, we treated FM cells with Novobiocin (BCRP inhibitor) to trap the eFFlux dye inside cell (green peak; Figure 3A). Cells with higher green fluorescence indicate functionally active BCRP. Cells with inactive BCRP allow efflux dye to escape the cell and subsequently lower fluorescence, indicating a lack of functionally relevant BCRP. This fluorescence intensity was measured as multidrug resistance (MDR) values. Compared to BeWo cells, there is a significant difference in the expression of multidrug resistance activity across FM cells (*p* < 0.001). These data suggest that BCRP has a differential functional response in FM cells along with BeWo at the maternal-fetal interface.

### 3.4. CTC- and BeWo-Derived Exosomes Package the BCRP Transport Protein as Cargo

**Figure 4:**
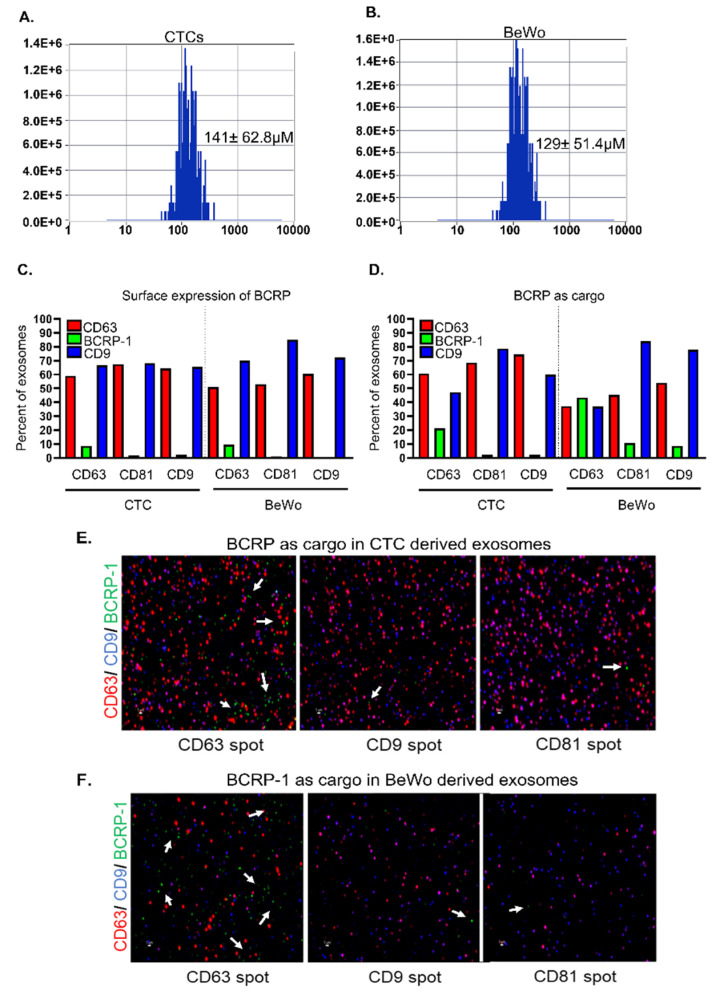
BeWo and CTC derived exosomes contain BCRP as cargo. Size distribution of (**A**) CTCs- and (**B**) BeWo-derived exosomes by ZetaView. (**C**) Surface localization of the BCRP protein in CTC- and BeWo-derived exosomes shown as a percentage of the total exosomes. The green bar represents the exosomes with BCRP on the surface, which also expresses either CD63, CD 81, or CD9. Limited surface expression was demonstrated in both the CTC and BeWo cells. (**D**) BCRP as cargo in CTC- and BeWo-derived exosomes shown as a percentage of the total exosomes. The green bar represents the exosomes that contain the BCRP protein as cargo, which also express either CD63, CD 81, or CD9. Exosomes that expressed CD63 contained the highest percentage of BCRP protein. Approximately 20% of the CTC-derived exosomes were CD63-BCRP positive. In contrast, approximately 40% of the BeWo-derived exosomes were CD63-BCRP positive. (**E**) BCRP protein detection with ExoView™ cargo staining in CTC-derived exosomes. CD63 (red), CD9 (blue), and BCRP (green- white arrows). (**F**) BCRP protein detection with ExoView™ cargo staining in BeWo-derived exosomes. CD63 (red), CD9 (blue), and BCRP (green-white arrows). Scale bar, 1 µm.

The analysis of CTC- and BeWo-derived exosomes using ZetaView^®^ demonstrated a size distribution of 120–140 nm, as shown in Figure 4A,B. The CTC-derived exosomes demonstrated an average size of 141 ± 62.8 nm, whereas the BeWo-derived exosomes demonstrated an average size of 129 ± 51.4 nm (Figure 4A,B). To test the hypothesis that CTC- and BeWo-derived exosomes package the drug transport protein BCRP, ExoView^®^ single particle interferometric reflectance imaging sensor (SP-IRIS) technology was utilized to characterize the exosomes. In both CTC- and BeWo-derived exosomes, BCRP was seen more often among those exosomes captured by the CD63 tetraspanin for both surface and luminal cargo expression compared to the other tetraspanin markers captured (Figure 4C,D). The green bars in Figure 4C,D represent the surface expression of BCRP in cell-derived exosomes calculated as a percentage of the total exosomes. The CTC- and BeWo-derived exosomes demonstrated the limited expression of BCRP on the surface of the exosomes, as shown in Figure 4C, with 10% of CD63 exosomes being positive for BCRP in CTC and 20% of CD63 exosomes being positive for BCRP in BeWo. To test the hypothesis that CTC- and BeWo-derived exosomes carry BCRP as cargo, the internal BCRP protein was detected and the results were calculated as a percentage of total exosomes using the ExoView^®^ cargo protocol. The green bars again represent the exosomes that contain the BCRP protein as cargo (Figure 4D). The exosomes captured on the CD63 spot contained the highest percentage of the BCRP protein as cargo and a 4-fold increase in the detection of the number of positive EVs compared to surface expression only, confirming that BCRP is primarily transported as cargo inside exosomes. In total, 43.4% of the BeWo-derived exosomes examined by ExoView^®^ contained BCRP as a cargo protein in the CD63 tetraspanin-enriched exosomes. This is twice the amount of CTC-derived exosomes, which constituted 21.1% of the total CTC exosomes examined. The same number of total exosomes were loaded into the assay for each sample based on ZetaView^®^ counting results. As far as we are aware, this is the first report that BCRP is carried as cargo by exosomes derived from CTC and BeWo cells. The data also support the fact that BCRP is localized both on the surface and in the lumen of these exosomes with greater expression in the lumen. These data suggest that BCRP carried in exosomes may function to control the local tissue environment. Although an interesting concept, the functional role of exosomal BCRP was beyond the scope of this report.

## 4. Discussion

Knowledge of drug transport during pregnancy is required to understand the pharmacokinetics of pregnancy. The potential mechanism of drug transport across the placenta and FM cannot be disregarded when designing preclinical and clinical trials studying drug pharmacokinetics during pregnancy. To test the hypothesis that FMs contribute to drug transport like the placenta, the expression of a drug transport protein on the FM and its cells was investigated. This study demonstrated that BCRP is expressed in FMs and is functional. The findings of this study are as follows: (1) ABCG2 is expressed in human FMs, (2) BCRP is localized in CTCs and AECs, (3) BCRP expressed in FM cells demonstrates efflux functions, and (4) along with tissue and cellular expression, FM and placenta cell-derived exosomes also express BCRP as cargo. These findings are significant and suggest that the FMs can also contribute to drug transport during pregnancy and protect the fetus by effluxing exogenous substances from the amniotic fluid to the maternal circulation.

Efflux transporter expression in the placenta were reported to decrease as gestation progresses [32,33]. Pathological changes in the placenta like ischemia and infarction can also lead to a decrease in BCRP expression resulting in less efflux of harmful xenobiotics [34,35]. Since the protection of the fetus from xenobiotic agents is paramount as gestation progresses, there should be an alternate efflux mechanism to protect the fetus. The data presented in this study support the hypothesis that the FMs could potentially compensate efflux activity by the placenta. It is also important to explore the regulation of transport proteins in FMs. This new knowledge could be used in drug development research to target drug transporters located on FM cells to enhance the therapeutic activity of drugs. The drug transport proteins located on the FM could possibly serve as a better target for drug development given their higher drug transport capability. To support the evidence that FMs participate in the drug transport mechanism, the efflux dye assay demonstrated the functional activity of the FM cells. The differential functional response of BCRP across FM cells demonstrated that AECs that are directly in contact with amniotic fluid followed by AMCs and CTCs can participate in the efflux of xenobiotics and drugs used during pregnancy like statins, glyburide, and sulfasalazine from the fetal to the maternal side. These novel discoveries impact our understanding of pharmacology during pregnancy and have the potential to affect the development and study of current and novel drug therapies used in pregnancy. Preliminary data from our laboratory demonstrated that infection and drug substrate stimulation do not cause a change in BCRP expression in CTC or BeWo cells (data not shown). Future studies are warranted to examine the response of transport proteins, like BCRP, to various stimuli, as they could affect the amount of a drug that crosses the FM and placenta cells to reach the fetus. 

As far as we are aware, this is the first report of BCRP expression and function in the human FM exosomes. BCRP was selected as the transport protein given its expression in multiple tissues throughout the body, the fact that it is a well-known protein of importance in drug transport, and because its expression can change depending on the microenvironment [36,37,38]. Besides the infection-regulating BCRP expression, other cellular processes could exert control during pregnancy [39]. Progesterone and 17b-estradiol hormones have been shown to down-regulate BCRP expression in BeWo cells [40] and hypoxia signaling could also down-regulate BCRP expression and efflux activity in BeWo cells [41]. Similarly, our study also showed that the expression of BCRP in amnion mesenchymal cells is very limited. This finding suggests that there might be a loss of BCRP expression during an epithelial-mesenchymal transition when amnion epithelial cells are transitioned to remodel FMs during pregnancy. These data also support our previous reports that amnion mesenchymal cells are just transient cells that will become epithelial cells again and do not stay within the extracellular matrix region of the membrane to perform any specific transport functions [23,42]. 

One of the strengths of our study is the investigation of BCRP in FM- and placenta-derived exosomes. The concept that drug transport proteins can be transported as cargo by FM and placenta exosomes is a novel concept that has not been previously reported. The principal findings of this study are that BCRP has limited expression on the surface of CTC- and BeWo-derived exosomes (9% and 9%, respectively). The lack of a BCRP protein on the surface of cell-derived exosomes is in congruence with the current understanding of the role of exosomes. Exosomes carry important cargo from their cell of origin to a recipient cell to help carry out specified functions [19]. The exosome itself does not bind drugs to carry out a function, but is rather a transporter of important cargo; therefore, the lack of BCRP on the surface of exosomes is expected. Our data demonstrate that the CTC- and BeWo-derived exosomes carry the BCRP protein as cargo (40% in BeWo and 20% in CTCs). Since exosomes play an important role in cellular communications during pregnancy [19], it is likely that drug transport by a cell is regulated via exosomes. The difference in the expression and function of these exosomes at the recipient cells warrants further investigation. This novel discovery could lead to the development of drug therapies that target exosomes carrying BCRP, especially the exosomes that tend to express the CD63 tetraspanin marker, as the class of exosomes with a higher CD63 demonstrated a higher percentage of BCRP as cargo. The exosomes that tend to express the tetraspanin marker CD63 are thought to play a role in loading transmembrane proteins into intraluminal vesicles [43]. Drug transport proteins are transmembrane proteins and could be packaged into these CD63 exosomes for transport to a target tissue to carry out a specific function. In addition, cells potentially shed and excrete BCRP as cargo via exosomes which can modify the tissue environment in a paracrine fashion. These exosomes could potentially make other cells perform transporter protein functions. The difference in the BeWo- and CTC-derived exosomes carrying BCRP as a cargo cannot be explained but will hopefully serve as a starting point for further studies investigating the role that exosomes potentially play in drug transportation. 

The use of BeWo cells as an in vitro model to study placenta is one limitation of our study. The commercially available BeWo cell line has been used in several previous studies as a proxy for placenta cells [40]. However, the question remains whether these cells derived from a choriocarcinoma accurately reflect drug transport in non-cancerous placental cells. Another limitation is the lack of functional studies evaluating BCRP in BeWo- and CTC-derived exosomes. The presence of BCRP in these exosomes does not necessarily translate to a functional change at the target tissue. Further studies are warranted to investigate whether these exosomes play a role in drug transport between the mother and developing fetus. Exosomes carrying BCRP as cargo could potentially carry the BCRP drug transport protein to cells that do not express BCRP, ultimately affecting the drug transport in these cells. 

Our study has multiple strengths and novel discoveries that could possibly change the field of obstetrics pharmacology. The discovery of the expression and localization of ABCG2/BCRP on the FM is an important breakthrough in the study of drug transport at the feto-maternal interface during pregnancy. Our hope is that this new understanding leads to improved preclinical and clinical drug trials with the goal of improving pharmacotherapy in pregnancy to improve maternal and neonatal outcomes.

## Data Availability

The raw data supporting the conclusion of this article will be made available by the authors, without undue reservation.
